# Predictive value of pretreatment albumin‐to‐alkaline phosphatase ratio for overall survival for patients with advanced non‐small cell lung cancer

**DOI:** 10.1002/cam4.3244

**Published:** 2020-07-21

**Authors:** Shaozhang Zhou, Wei Jiang, Huilin Wang, Ni Wei, Qitao Yu

**Affiliations:** ^1^ Department of Respiratory Oncology Guangxi Medical University Affiliated Tumor Hospital Nanning City Guangxi Zhuang Autonomous Region China; ^2^ Department of No.5 Chemotherapy Guangxi Medical University Affiliated Tumor Hospital Nanning City Guangxi Zhuang Autonomous Region China

**Keywords:** AAPR, association, NSCLC, overall survival, subgroup analysis

## Abstract

**Objectives:**

To investigate the relation between AAPR and OS in patients with advanced non‐small cell lung cancer (NSCLC).

**Methods:**

A retrospective cohort study was conducted with 808 patients with advanced NSCLC who were treated in Guangxi Medical University Affiliated Tumor Hospital in China from 5 March 2009 to 31 August 2018. The target‐independent and dependent variables were AAPR measured in patients before anticancer treatment and overall survival (OS), respectively. Covariates involved in this study included age, gender, ECOG status, smoking history, clinical stages, pathological type, driver mutation (EGFR or ALK), metastasis or not (bone, lung, liver, brain, malignant plural effusion, and other organs), number of organ metastasis(≤3, >3), first‐line regiment and number of treatment lines (≤3, >3).

**Results:**

The mean age of the selected patients was 58.3 ± 10.9 years and 68.6% were male. We divided patients according to their AAPR into low (AAPR < 0.34, n = 266), medium (AAPR = 0.34‐0.47, n = 259), and high (AAPR > 0.47, n = 283) tertile groups. Medium and high AAPR were associated with a decreased risk of death after fully adjusted Cox proportional hazard model(s) with hazards ratio (HR) 0.77 (95%CI = 0.58‐1.03) and HR 0.59 (95%CI = 0.45‐0.78), respectively (*P* for trend <.05). The median OS of low, medium, and high AAPR was 9.3, 11.8, and 16.9 months, respectively (*P* value <.0001). No optimal cutoff value of AAPR for prognosing OS was identified by smooth curve fitting. The HR and the 95% confidence intervals of the left and right sides of the inflection point 0.6 as cutoff value were 0.28 (95%CI = 0.14‐0.57) and 0.77 (95%CI = 0.34‐1.73), respectively (*P* value = .127). By subgroup analysis, similar results were consistently observed across nearly all the subgroups.

**Conclusion:**

Our study implied that pretreatment AAPR can be used as an independent prognostic factor in patients with advanced NSCLC. This ratio should be applied for risk stratification and clinical decision‐making in those patients.

## Graphical abstract

Using a retrospective cohort study with a large number of patients, this study investigates the relationships between albumin‐to‐alkaline phosphatase ratio as a prognostic factor for patients with advanced non‐small cell lung cancer.

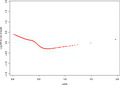

## 1 INTRODUCTION

Lung cancer remains the most diagnosed cancer type, which accounts for approximately 20% of cancer‐related mortality worldwide.^1^, ^2^ Nearly 80% of patients present with locally advanced or metastatic disease at the time of diagnosis. Over the past decades, treatments for advanced NSCLC were confined to platinum‐based chemotherapy with a modest response rate and a median overall survival (OS) of 30%‐35% and 10‐11 months, respectively.^3^ Although targeted therapy and immunotherapy implemented in clinical practice have improved clinical outcomes dramatically in recent years, the 5‐year OS is still unsatisfactory. The TNM staging system of American Joint Committee on Cancer (AJCC), a widely used staging system, shows reliable and stable predicting abilities for most cancer types. However, the TNM staging system appears to reach limitations when discriminate outcomes for patients with advanced stage cancers. It is of great importance as well as a necessity to explore the new survival prediction markers that could be used for risk stratification and clinical decision‐making for patients with advanced NSCLC.

Various serum markers, which can be obtained rapidly, conveniently, and repeatedly, have been developed to predict prognosis. The use of clinicopathological features combined with serum markers as prognostic indicators for predicting outcomes for variety of cancer types has been validated in a substantial number of publications in recent years. The serum markers include: neutrophil‐to‐lymphocyte ratio (NLR),^4^ platelet to lymphocyte ratio (PLR),^5^ hemoglobin‐to‐red cell distribution width,^6^ and other markers. In this study, we combined two laboratory parameters: albumin (ALB) with alkaline phosphatase (ALP) to create a novel prognostic index called albumin‐to‐alkaline phosphatase ratio (AAPR). It is now well‐established from a variety of studies, that AAPR has been examined in several cancer types showing some promising results.^7^, ^8^, ^9^, ^10^ However, few researchers have addressed the relationship between AAPR and OS in patients with advanced NSCLC. Therefore, in this work, we conducted a retrospective study with a large cohort of patients aiming to analyze the prognostic power of AAPR in advanced NSCLC.

## 2 MATERIALS AND METHODS

### 2.1 Study design

To explore the relationship between AAPR and OS in patients with advanced NSCLC after adjusting for the potential confounders, we conducted a retrospective cohort study. The target‐independent variable AAPR was obtained at the baseline level before any anticancer treatment. The dependent variable was OS (dichotomous variable: 1 = death; 0 = alive).

### 2.2 Patients

The entire process of data collection was nonselective and consecutive. The data of patients with advanced NSCLC who were admitted to Guangxi Medical University Affiliated Tumor Hospital, Guangxi Province, China were collected. The identifiable information of patients was unnamed or anonymous with the aim to protect patients' privacy. Data are stored in electronic data acquisition system. Patients' informed consent was not required because of the nature of retrospective cohort study. This study was approved by the ethics committee of the hospital.

Patients' entry time and deadline for inclusion were 5 March 2009 and 31 August 2018, respectively. A total of 1076 patients were initially enrolled in this study for further screening. Inclusion criteria were as follows: (a) Patients with either histologically or cytologically confirmed diagnosis of advanced NSCLC; (b) Sociodemographic, clinicopathological characteristics, and complete follow‐up information of all patients were available; (c) Patients were not receiving any anticancer therapies at the time of initial diagnosis; (d) There was no concurrent malignancy or a history of a second primary malignancy. In addition, patients with concurrent liver disease, including liver cirrhosis, and those with confirmed hepatitis B or C virus infection, that could affect AAPR levels, were excluded. Patients with small cell lung cancer or staged with not advanced NSCLC or incomplete baseline information were also excluded.

### 2.3 Variables

We obtained pretreatment AAPR at baseline and recorded it as a continuous variable. The detailed process is described as follows: values for ALB and ALP at baseline levels before treatment were extracted from electronic medical records. The AAPR was calculated by dividing the serum ALB level by the serum ALP level.

Final outcome variable (dichotomous variable), the OS, was calculated from the date of diagnosis of advanced NSCLC to the date of patient death or a last follow‐up which was obtained from the information on regular follow‐ups.

Covariates involved in the present study can be summarized as follows: (a) demographic data; (b) variables that can potentially affect AAPR or OS as reported by previous studies; (c) additional variables based on our clinical experiences. Therefore, the following variables were used to construct the fully adjusted model: (a) continuous variable: age (obtained at baseline); (b) categorical variables: gender, Eastern Cooperative Oncology Group (ECOG) status, smoking history, clinical stages (IIIB or IV), pathological type, driver mutation (epidermal growth factor receptor (EGFR) or anaplastic lymphoma kinase (ALK)), metastasis or not (bone, lung, liver, brain, malignant plural effusion, and other organs), number of organ metastasis (≤3 or >3), and number of treatment lines (≤3 or >3) (obtained at baseline).

The definitions of other clinicopathological characteristics or parameters that we used in this study included the following: patients' physical status was scored by ECOG‐performance status (ECOG‐PS). Patients who had smoked no more than 100 cigarettes in their lifetime were defined as nonsmokers. Smokers were defined as current smokers or individuals who had stopped smoking for less than 1 year before diagnosis. Tumor histology was classified according to the 3rd edition of WHO Classification of tumors. Tumor stages were determined using current AJCC guidelines (version 7th edition).

### 2.4 Follow‐up procedure

The follow‐up was performed by the first four authors of this study. The cutoff date for patients' follow‐up was 31 August 2019. Data were stored in the follow‐up system provided by the hospital. Follow‐up interval was every 3 months.

### 2.5 Statistical analysis

In this study, continuous variables in case of a normal distribution were expressed as mean ± standard, and for other cases as moderate (min, max). Categorical variables were expressed in frequency or as a percentage. Chi‐squared test (categorical variables), Student *t* test (normal distribution), or Mann‐Whitney *U* test (skewed distribution) was used to test for differences among different AAPR groups. The data analysis process of this study was based on the following three criteria: (a) what is the relationship between AARP and OS (linear or non‐linear); (b) which factors modify or interfere with the relationship between AARP and OS; and (c) adjustment of the interference factors or after the stratified analysis, what is the true relationship between AARP and OS? Therefore, data analysis can be summarized in three steps. Step 1: Univariate and multivariate Cox proportional hazard model(s) were built. We constructed three models, namely model 1, no covariates were adjusted; model 2, adjusted only for sociodemographic data; and model 3, model 2+ other covariates presented in Table 1. Step 2: To address the nonlinearity between AAPR and OS, a Cox proportional hazards ratio (HR) model with cubic spline functions and smooth curve fitting (penalized spline method) were performed. If nonlinearity was found, we first calculated the inflection point using recursive algorithm, and then constructed a two‐piecewise Cox proportional hazard model(s) on both sides of the inflection point. Step 3: The subgroup analyses were conducted using stratified Cox proportional hazard model(s). Overall survival among groups was first assessed using the Kaplan‐Meier method and log‐rank tests. All the analyses were performed with the statistical software packages R (http://www.R‐project.org, The R Foundation) and EmpowerStats (http://www.empowerstats.com, X&Y Solutions, Inc, Boston, MA). All tests were two‐sided and *P* values lower than .05 were considered statistically significant.

**TABLE 1:** The relationship between AAPR tertile and clinicopathological parameters in the present advanced NSCLC cohort (n = 808)

AAPR Tertile	Total	Low	Medium	High	*P*‐value
Number of patients	808	266	259	283	
Age	58.32 ± 10.88	57.91 ± 9.73	59.47 ± 10.68	57.67 ± 11.97	.117
Age					.076
<65	577 (71.41%)	203 (76.60%)	177 (68.34%)	197 (69.61%)	
≥65	231 (28.59%)	62 (23.40%)	82 (31.66%)	86 (30.39%)	
Gender					<.001
Male	556 (68.64%)	193 (72.56%)	193 (74.52%)	170 (60.07%)	
Female	254 (31.36%)	73 (27.44%)	66 (25.48%)	113 (39.93%)	
Smoking history					.061
Never	385 (47.59%)	119 (44.74%)	110 (42.64%)	154 (54.42%)	
Ever	413 (51.05%)	144 (54.14%)	144 (55.81%)	125 (44.17%)	
Unknown	11 (1.36%)	3 (1.13%)	4 (1.55%)	4 (1.41%)	
ECOG					.023
0‐1	655 (87.45%)	202 (82.79%)	213 (89.87%)	239 (89.85%)	
≥2	94 (12.55%)	42 (17.21%)	24 (10.13%)	27 (10.15%)	
Pathology					.090
Adenocarcinoma	593 (73.21%)	211 (79.32%)	178 (68.73%)	203 (71.73%)	
Squamous cell carcinoma	190 (23.46%)	48 (18.05%)	71 (27.41%)	70 (24.73%)	
Others	27 (3.33%)	7 (2.63%)	10 (3.86%)	10 (3.53%)	
Clinical stage					<.001
IIIA + IIIB	120 (14.81%)	20 (7.52%)	49 (18.92%)	51 (18.02%)	
IV	690 (85.19%)	246 (92.48%)	210 (81.08%)	232 (81.98%)	
Bone					<.001
No	506 (64.05%)	117 (44.15%)	177 (68.87%)	210 (78.95%)	
Yes	284 (35.95%)	148 (55.85%)	80 (31.13%)	56 (21.05%)	
Liver					<.001
No	673 (85.19%)	202 (76.23%)	236 (91.83%)	234 (87.97%)	
Yes	117 (14.81%)	63 (23.77%)	21 (8.17%)	32 (12.03%)	
Lung					.578
No	489 (61.90%)	168 (63.40%)	162 (63.04%)	158 (59.40%)	
Yes	301 (38.10%)	97 (36.60%)	95 (36.96%)	108 (40.60%)	
Brain					.234
No	646 (81.77%)	209 (78.87%)	210 (81.71%)	225 (84.59%)	
Yes	144 (18.23%)	56 (21.13%)	47 (18.29%)	41 (15.41%)	
Pleural effusion					.581
No	491 (62.15%)	170 (64.15%)	160 (62.26%)	159 (59.77%)	
Yes	299 (37.85%)	95 (35.85%)	97 (37.74%)	107 (40.23%)	
Number of organ metastasis					<.001
≤3	429 (54.30%)	110 (41.51%)	140 (54.47%)	178 (66.92%)	
>3	361 (45.70%)	155 (58.49%)	117 (45.53%)	88 (33.08%)	
EGFR mutation					.183
Negative	205 (25.31%)	74 (27.82%)	66 (25.48%)	64 (22.61%)	
Positive	137 (16.91%)	53 (19.92%)	42 (16.22%)	42 (14.84%)	
Unknown	468 (57.78%)	139 (52.26%)	151 (58.30%)	177 (62.54%)	
ALK rearrangement					.001
Negative	300 (37.04%)	115 (43.23%)	96 (37.07%)	88 (31.10%)	
Positive	29 (3.58%)	16 (6.02%)	7 (2.70%)	6 (2.12%)	
Unknown	481 (59.38%)	135 (50.75%)	156 (60.23%)	189 (66.78%)	
First‐line regiment					.461
Platinum‐based doublet chemotherapy	411 (65.24%)	129 (63.55%)	127 (65.46%)	153 (66.23%)	
Single drug chemotherapy	36 (5.71%)	9 (4.43%)	16 (8.25%)	11 (4.76%)	
Targeted therapy	125 (19.84%)	49 (24.14%)	33 (17.01%)	43 (18.61%)	
Platinum‐based doublet chemotherapy plus angiogenesis‐therapy	49 (7.78%)	14 (6.90%)	14 (7.22%)	21 (9.09%)	
Others	9 (1.43%)	2 (0.99%)	4 (2.06%)	3 (1.30%)	
Number of treatment lines					.811
≤3	489 (76.89%)	161 (78.54%)	150 (76.14%)	177 (76.29%)	
>3	147 (23.11%)	44 (21.46%)	47 (23.86%)	55 (23.71%)	

Abbreviations: AAPR, albumin‐to‐alkaline phosphatase ratio; ALK, anaplastic lymphoma kinase; ECOG‐PS, performance status of East Cooperative Oncology Group; EGFR, epidermal growth factor receptor; NSCLC, non‐small cell lung cancer.

## 3 RESULTS

### 3.1 Baseline characteristics of selected patients

**FIGURE 1:**
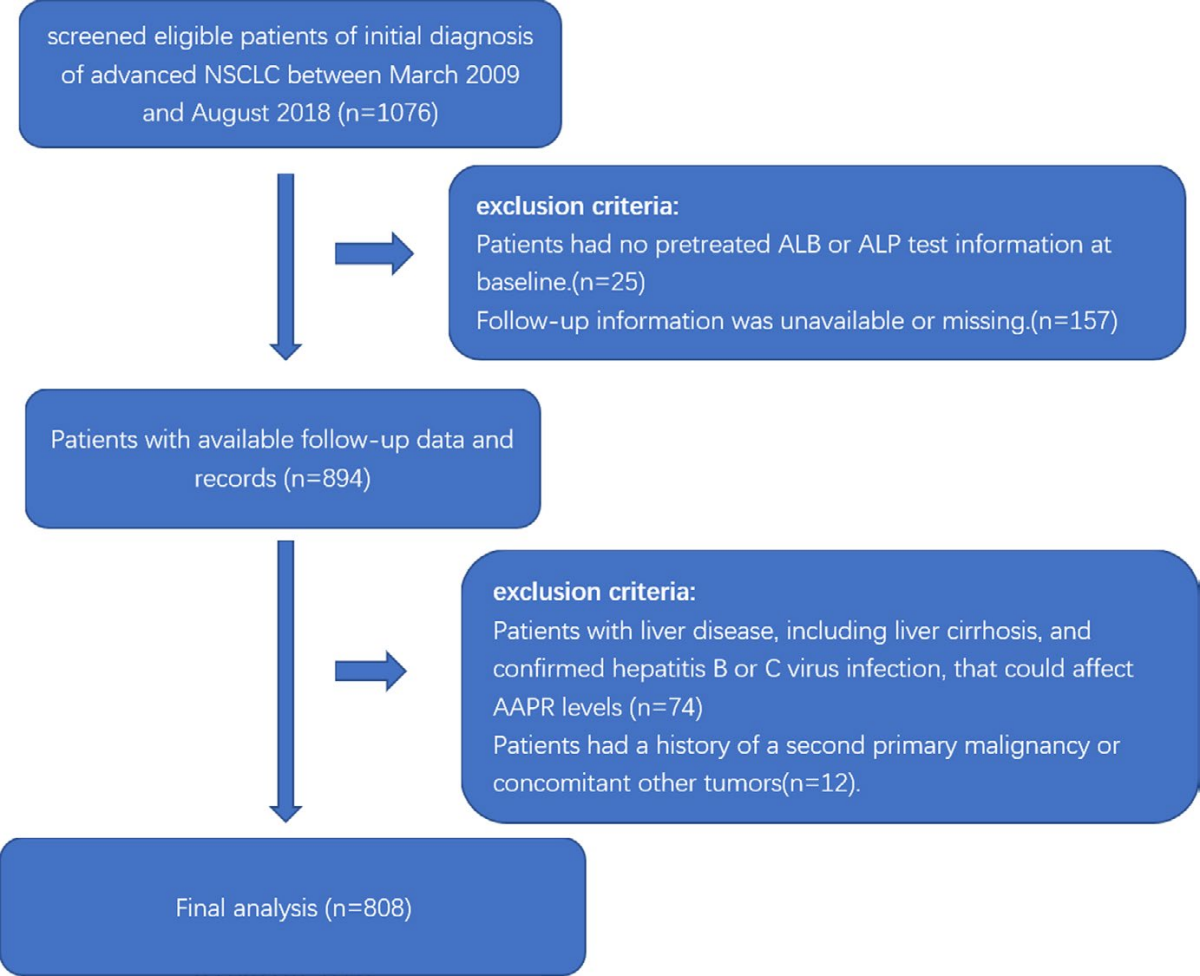
Flowchart for patients’ screening

Based on the inclusion and exclusion criteria, 808 patients were selected for the final data analysis (see Figure 1 for the flow chart). We divided AAPR tertile into low (AAPR < 0.34, n = 266), medium (AAPR = 0.34‐0.47, n = 259), and high (AAPR > 0.47, n = 283) groups. Table 1 shows baseline characteristics of the selected patients. Briefly, the average age of the 808 selected patients was 58.3 ± 10.9 years old with 68.6% of male patients. No statistically significant differences were found either in age (as contiguous and dichotomies variable), smoking history, pathology, metastasis in lung, brain, and pleural effusion, or in EGFR mutation among different AAPR groups with *P* values >.05 for all of these variables. There were statistically significant differences among three groups in gender, ECOG‐PS, clinical stage, metastases in bone and liver, number of organs affected by metastases, and ALK rearrangement with *P* value <.05 for all of these variables.

### 3.2 Univariate analysis

We listed the results of univariate analyses in Table 2. According to the results of univariate Cox proportional hazard model(s), we identified the following parameters to have an unfavorable prognosis with the increased risk of death: age ≥65, being male, a smoking history, ECOG‐PS ≥ 2, squamous cell carcinoma, stage IV, and metastases in bone, liver, and lung, whereas the opposite results were found in patients carrying EGFR mutation, ALK rearrangement, or with number of treatment lines >3.

**TABLE 2:** Prognostic factors for overall survival of advanced NSCLC patients in univariate Cox regression analyses

Covariates	Statistics	Status
AAPR	0.84 ± 0.36	1.41 (1.11, 1.78) 0.0048
Age		
<65	577 (71.41%)	1.0
≥65	231 (28.59%)	1.25 (1.04, 1.51) 0.0182
Gender		
Male	556 (68.64%)	1.0
Female	254 (31.36%)	0.80 (0.67, 0.96) 0.0187
Smoking history		
Never	385 (47.59%)	1.0
Ever	413 (51.05%)	1.40 (1.17, 1.66) 0.0002
Uncertain	11 (1.36%)	4.06 (2.21, 7.45) <0.0001
ECOG‐PS		
0‐1	655 (87.45%)	1.0
≥2	94 (12.55%)	1.52 (1.18, 1.95) 0.0011
Clarification		
Adenocarcinoma	593 (73.21%)	1.0
Squamous cell carcinoma	190 (23.46%)	1.38 (1.12, 1.69) 0.0021
Others	27 (3.33%)	1.35 (0.88, 2.07) 0.1749
Clinical stage		
IIIB	120 (14.81%)	1.0
IV	690 (85.19%)	1.30 (1.01, 1.67) 0.0418
Bone		
No	506 (64.05%)	1.0
Yes	284 (35.95%)	1.14 (0.95, 1.36) 0.1568
Liver		
No	673 (85.19%)	1.0
Yes	117 (14.81%)	1.31 (1.03, 1.66) 0.0286
Lung		
No	489 (61.90%)	1.0
Yes	301 (38.10%)	1.04 (0.87, 1.25) 0.6563
Brain		
No	646 (81.77%)	1.0
Yes	144 (18.23%)	1.11 (0.89, 1.39) 0.3479
Pleural effusion		
No	491 (62.15%)	1.0
Yes	299 (37.85%)	0.95 (0.80, 1.14) 0.6042
Number of metastatic organs		
≤3	429 (54.30%)	1.0
>3	361 (45.70%)	1.27 (1.07, 1.51) 0.0064
EGFR		
Negative	205 (25.31%)	1.0
Positive	137 (16.91%)	0.58 (0.43, 0.79) 0.0005
Unknown	468 (57.78%)	1.15 (0.94, 1.42) 0.1770
ALK		
Negative	300 (37.04%)	1.0
Positive	29 (3.58%)	0.73 (0.42, 1.26) 0.2531
Unknown	481 (59.38%)	1.20 (1.00, 1.44) 0.0509
First‐line regiment		
Platinum‐based doublet chemotherapy	411 (65.24%)	1.0
Single drug chemotherapy	36 (5.71%)	0.97 (0.64, 1.47) 0.8741
Targeted therapy	125 (19.84%)	0.96 (0.75, 1.24) 0.7588
Platinum‐based doublet chemotherapy plus angiogenesis‐therapy	49 (7.78%)	0.87 (0.58, 1.32) 0.5175
Others	9 (1.43%)	0.00 (0.00, Inf) 0.9888
Number of treatment lines		
≤3	489 (76.89%)	1.0
>3	147 (23.11%)	0.59 (0.46, 0.74) <0.0001

### 3.3 The nonlinear relationship between AAPR and the HR of the risk of death

**FIGURE 2:**
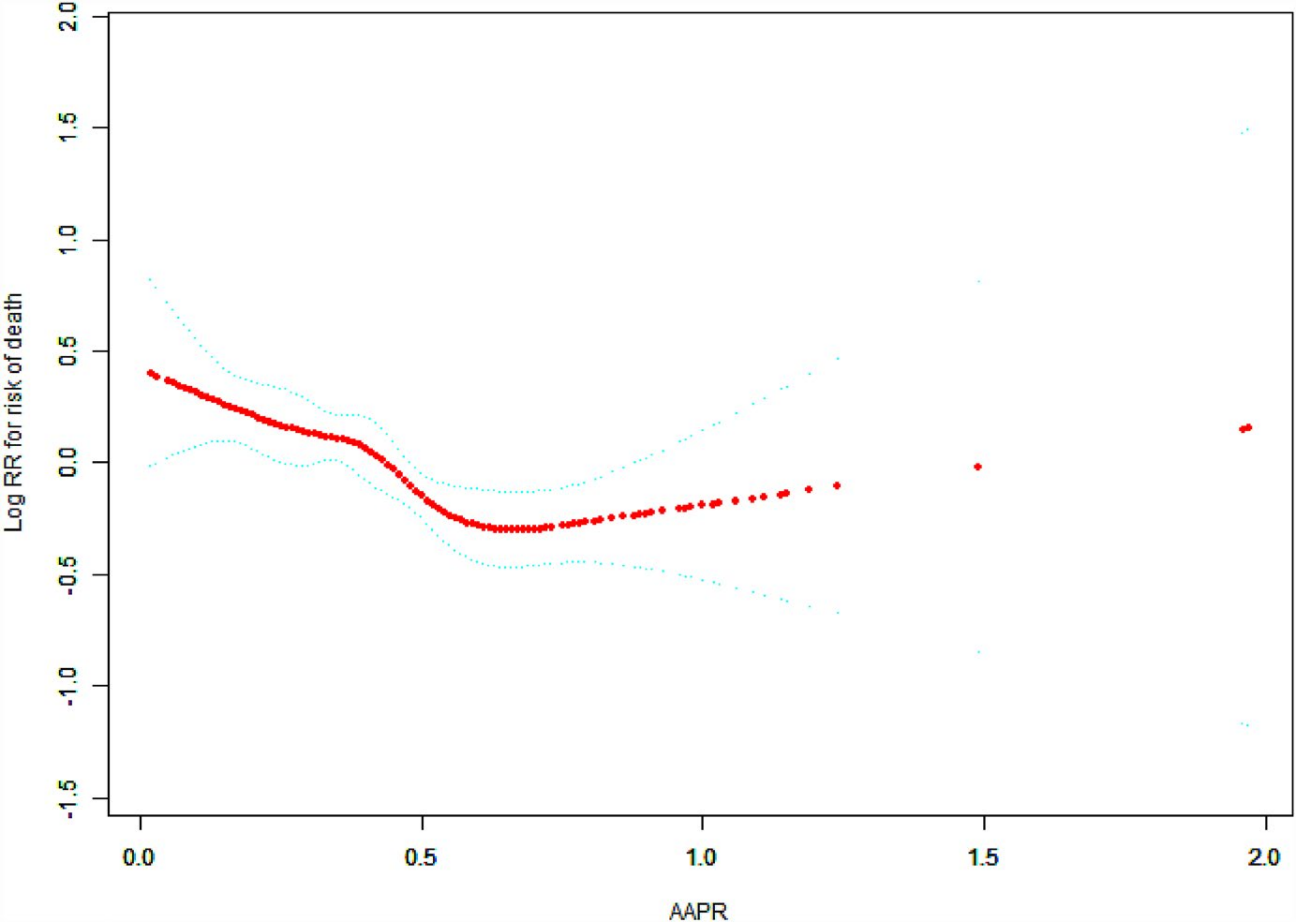
The relationships between the AAPR and the hazard ratio of the risk of death in patients with advanced NSCLC/Spline smoothing was performed using GAM (generalized additive model) to explore the association between AAPR and OS in patients with advanced NSCLC after adjusting for potential confounding factors. A nonlinear relationship between AAPR and OS was observed and a inflection point for AAPR of 0.6 was detected. The red points represent the fitting spline. The blue points represent the 95% confidence intervals

In this study, we analyzed the nonlinear relationship between AAPR and the risk of death (Figure 2). Smooth curve and the result of Cox proportional hazard model(s) with cubic spline functions after fully adjusting for potential variables used in this study showed that the relationship between AAPR and the HR for risk of death was linear (listed in Table 1). We used two‐piecewise Cox proportional hazard model(s) to fit the association between AAPR and the HR for risk of death based on *P* for log likelihood ratio test. By two‐piecewise Cox proportional hazard model(s) and recursive algorithm, we tested the saturation effect between AAPR and HR for OS with inflection point of 0.6. On the left side of the inflection point, the effect size and 95% CI were 0.28 and 0.14‐0.57, respectively. On the right side of the inflection point, the effect size and 95% CI were 0.77 and 0.34‐1.73, respectively (Table 3). Both saturation effect and threshold effect cannot be found using 0.6 as cutoff value in the study with *P* for log likelihood ratio test >.05.

**TABLE 3:** Threshold effect analysis of AAPR on OS using piecewise linear regression

Cutoff point of AAPR	HR (95% CI)^a^	*P* value
<0.6	0.28 (0.14, 0.57)	.0004
>0.6	0.77 (0.34, 1.73)	.5272
HR between <0.6 and >0.6	2.74 (0.77, 9.73)	.1197
Log Likelihood Ratio Test		.127

Note

An inflection point for the AAPR existed for overall survival in patients with advanced NSCLC. When AAPR was below the inflection point 0.6, HR decreased with the increase of AAPR level (HR = 0.29, 95% CI = 0.16‐0.52, *P* < .0001). When AAPR level exceeded to 0.6, the change in HR was not statistically significant (*P* > .05).

^a^ Adjusted: age, gender, clinical stage, smoking history, ECOG‐PS, pathology, liver metastasis, lung metastasis, bone metastasis, brain metastasis, malignant pleural effusion, number of metastatic organs, EGFR mutation status, ALK mutation status, number of treatment lines, first‐line regiments

### 3.4 Results of unadjusted and adjusted Cox proportional hazard model(s)

In this study, we constructed three models to analyze the independent effect of AAPR on OS (univariate and multivariate Cox proportional hazard model(s)). The effect sizes (HR) and 95% confidence intervals are listed in Table 4. HR of 0.79 and 0.59 for OS in fully adjusted model means that when compared with low AAPR group, the medium and high AAPR are associated with decreased 21% (HR = 0.79, 95%CI = 0.59‐1.06) and 41% (HR = 0.59, 95%CI = 0.44‐0.79) for risk of death, respectively. We also found the trends in the effect size in moderate and high AAPR groups were equidistant with approximate decrease of 20% (*P* value for trend = .0003).

**TABLE 4:** Multiple Cox regression analysis of AAPR in patients with advanced NSCLC

AAPR	N	With outcomes N (%)	Nonadjusted	*P* value	Adjust I	*P* value	Adjust II	*P* value
Continuous	808	529	0.44 (0.28, 0.67)	.0002	0.47 (0.29, 0.74)	.0014	0.52 (0.30, 0.88)	.0151
Tertile								
Low	266	187 (70.3%)	1.0		1.0		1.0	
Medium	259	160 (61.8%)	0.81 (0.66, 1.00)	.0525	0.77 (0.61, 0.97)	.0277	0.77 (0.58, 1.03)	.0756
High	283	182 (64.3%)	0.63 (0.51, 0.77)	<.0001	0.65 (0.52, 0.81)	.0001	0.59 (0.45, 0.78)	.0001
*P* trend			0.23 (0.12, 0.45)	<.0001	0.26 (0.13, 0.53)	.0002	0.20 (0.08, 0.45)	.0001

Note

Nonadjusted model adjusted for: None.

Adjust I model adjusted for: age, gender, clinical stage, smoking history, ECOG‐PS, pathology.

Adjust II model adjusted for: age, gender, clinical stage, smoking history, ECOG‐PS, pathology, liver metastasis, lung metastasis, bone metastasis, brain metastasis, malignant pleural effusion, number of metastatic organ, number of treatment lines, EGFR mutation status, ALK mutation status, first‐line regiments.

**FIGURE 3:**
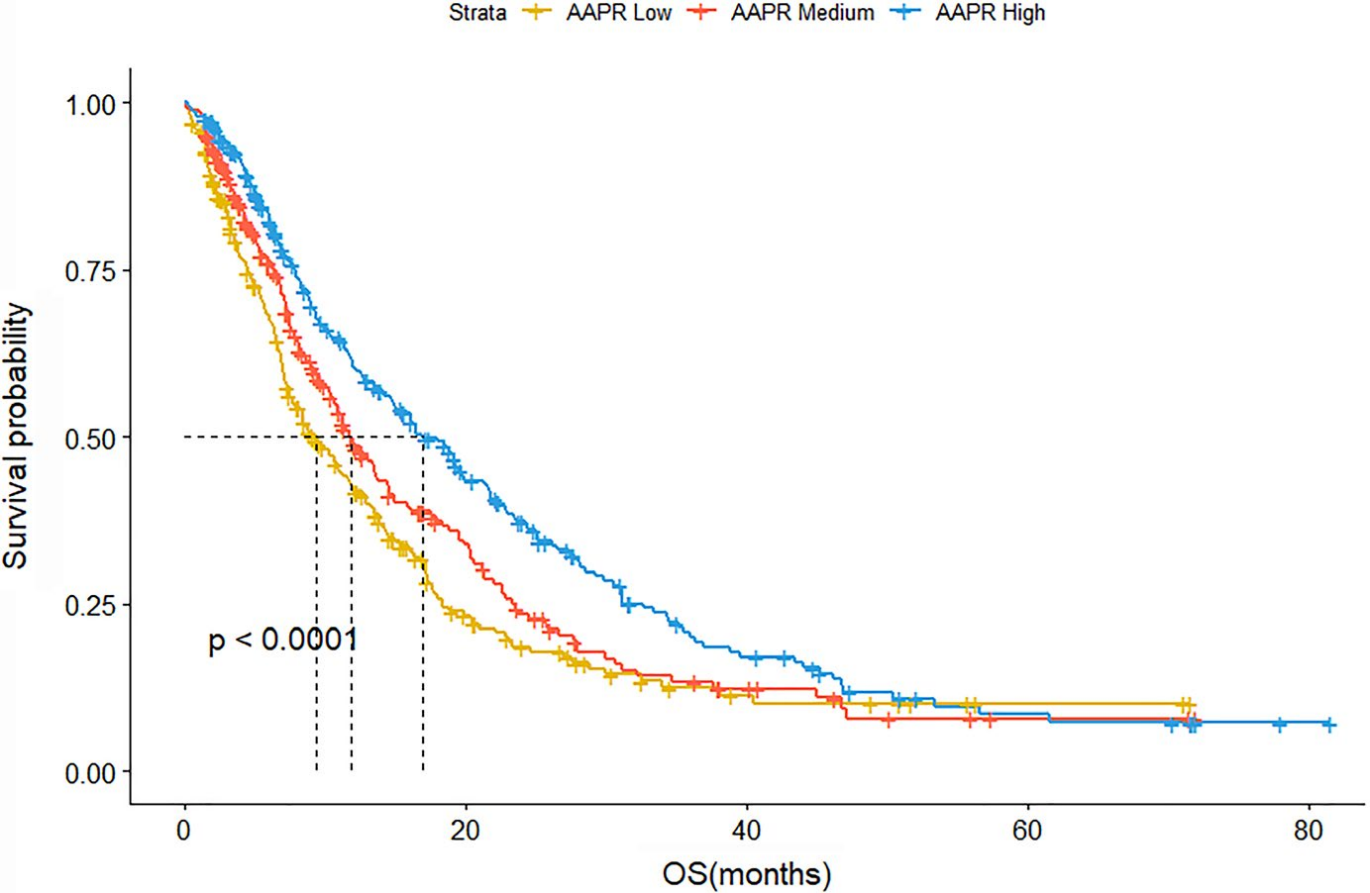
Kaplan‐Meier curves of overall survival in patients with NSCLC stratified by AAPR low, medium, and high groups

Figure 3 shows the Kaplan‐Meier curves of overall survival in patients with NSCLC stratified by AAPR groups. The median OS in low, medium, and high AAPR groups was 9.3 (95%CI = 7.5‐11.8), 11.8 (95%CI = 10.4‐14.5), and 16.9 (95%CI = 14.7‐21.6) months, respectively. These differences between groups were statistically significant (log‐rank test, *P* = .0001).

### 3.5 Subgroup analysis

**FIGURE 4:**
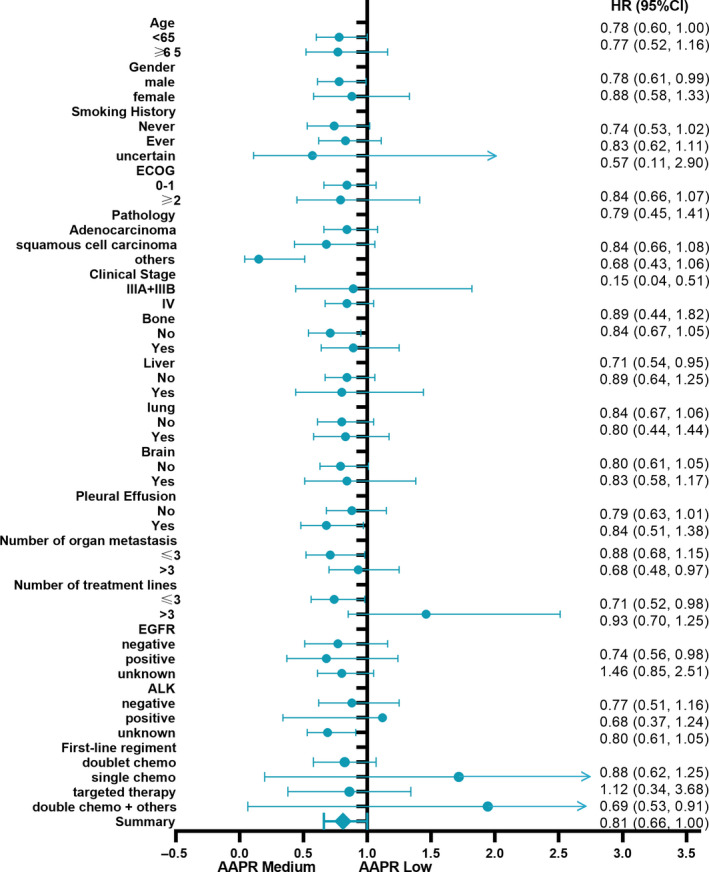
Forest plot for presenting the association between the hazard ratio of overall survival and medium AAPR in advanced NSCLC patients

**FIGURE 5:**
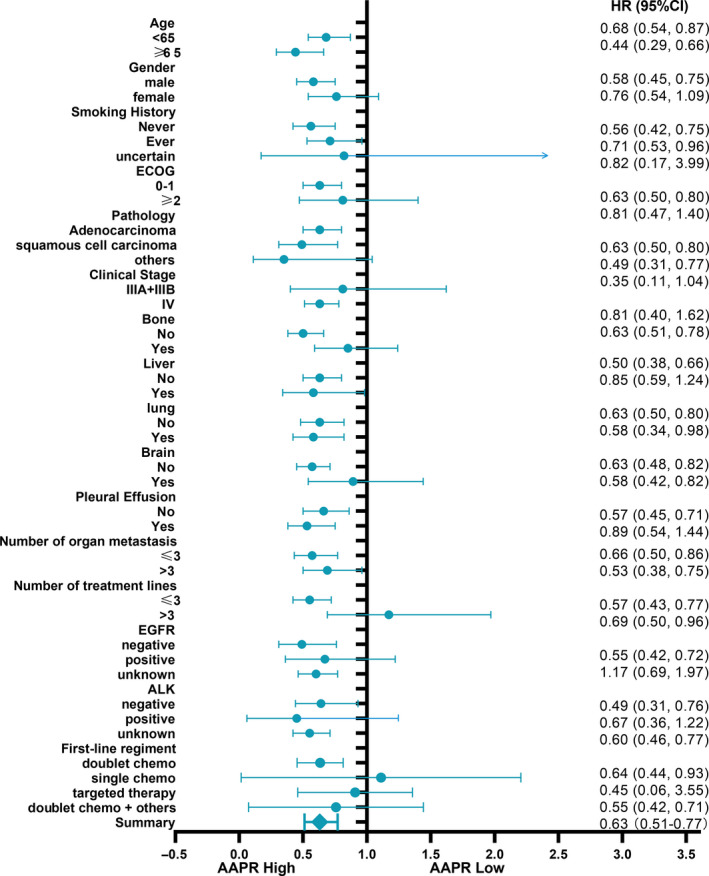
Forest plot for presenting the association between the hazard ratio of overall survival and high AAPR in advanced NSCLC patients

To further confirm that the findings presented in Table 5 are robust to potential confounders, we conducted stratified analyses by subgroups defined by covariables listed in Table 1. Age (<65, ≥65), gender, ECOG‐PS, smoking history, clinical stages (III or IV), pathological type, driver mutation (EGFR or ALK), metastases or not (bone, lung, liver, brain, malignant plural effusion, and other organs), number of organ metastases (≤3 or >3), and treatment lines (≤3 or >3) were stratified (Table 5). Figures 4 and 5 reveals a highly consistent pattern: medium and high AAPR values that can serve as an independent favorable prognostic indicator in advanced NSCLC were observed across nearly all the subgroups except for patients with number of treatment lines >3 or a positive status for ALK rearrangement (both *P* value >.05). The former (number of treatment lines >3) indicated that unfavorable outcome may be related to overtreatment and the latter (positive status for ALK rearrangement) can be explained by the small patient number after stratification (n = 29).

**TABLE 5:** Subgroup analysis using potential confounders as the stratification variables

AAPR Tertile	N	Low	Medium	*P* value	High	*P* value	*P* value for tread
Age							
<65	577	1.0	0.78 (0.60, 1.00)	.0477	0.68 (0.54, 0.87)	.0020	.002
≥65	231	1.0	0.77 (0.52, 1.16)	.2109	0.44 (0.29, 0.66)	<.0001	<.0001
Gender							
Male	556	1.0	0.78 (0.61, 0.99)	.0433	0.58 (0.45, 0.75)	<.0001	<.0001
Female	252	1.0	0.88 (0.58, 1.33)	.5479	0.76 (0.54, 1.09)	.1355	.1330
Smoking history							
Never	383	1.0	0.74 (0.53, 1.02)	.0637	0.56 (0.42, 0.75)	<.0001	<.0001
Ever	413	1.0	0.83 (0.62, 1.11)	.2058	0.71 (0.53, 0.96)	.0265	.0260
Uncertain	11	1.0	0.57 (0.11, 2.90)	.4957	0.82 (0.17, 3.99)	.8012	.8608
ECOG							
0‐1	654	1.0	0.84 (0.66, 1.07)	.1646	0.63 (0.50, 0.80)	.0001	<.0001
≥2	93	1.0	0.79 (0.45, 1.41)	.4328	0.81 (0.47, 1.40)	.4601	.4359
Pathology							
Adenocarcinoma	592	1.0	0.84 (0.66, 1.08)	.1825	0.63 (0.50, 0.80)	.0002	.0002
Squamous cell carcinoma	189	1.0	0.68 (0.43, 1.06)	.0893	0.49 (0.31, 0.77)	.0021	.0023
Others	27	1.0	0.15 (0.04, 0.51)	.0024	0.35 (0.11, 1.04)	.0592	.2482
Clinical stage							
IIIA + IIIB	120	1.0	0.89 (0.44, 1.82)	.7579	0.81 (0.40, 1.62)	.5516	.5338
IV	688	1.0	0.84 (0.67, 1.05)	.1308	0.63 (0.51, 0.78)	<.0001	<.0001
Bone							
No	504	1.0	0.71 (0.54, 0.95)	.0191	0.50 (0.38, 0.66)	<.0001	<.0001
Yes	284	1.0	0.89 (0.64, 1.25)	.5057	0.85 (0.59, 1.24)	.4104	.3665
Liver							
No	672	1.0	0.84 (0.67, 1.06)	.1415	0.63 (0.50, 0.80)	<.0001	<.0001
Yes	116	1.0	0.80 (0.44, 1.44)	.4546	0.58 (0.34, 0.98)	.0434	.0423
Lung							
No	488	1.0	0.80 (0.61, 1.05)	.1026	0.63 (0.48, 0.82)	.0007	.0007
Yes	300	1.0	0.83 (0.58, 1.17)	.2821	0.58 (0.42, 0.82)	.0018	.0017
Brain							
No	644	1.0	0.79 (0.63, 1.01)	.0557	0.57 (0.45, 0.71)	<.0001	<.0001
Yes	144	1.0	0.84 (0.51, 1.38)	.4905	0.89 (0.54, 1.44)	.6247	.6128
Pleural effusion							
No	489	1.0	0.88 (0.68, 1.15)	.3598	0.66 (0.50, 0.86)	.0019	.0019
Yes	299	1.0	0.68 (0.48, 0.97)	.0315	0.53 (0.38, 0.75)	.0003	.0004
Number of organ metastasis							
≤3	428	1.0	0.71 (0.52, 0.98)	.0343	0.57 (0.43, 0.77)	.0002	.0002
>3	360	1.0	0.93 (0.70, 1.25)	.6426	0.69 (0.50, 0.96)	.0277	.0333
EGFR							
Negative	204	1.0	0.77 (0.51, 1.16)	.2133	0.49 (0.31, 0.76)	.0017	.0016
Positive	137	1.0	0.68 (0.37, 1.24)	.2069	0.67 (0.36, 1.22)	.1897	.1696
Unknown	467	1.0	0.80 (0.61, 1.05)	.1158	0.60 (0.46, 0.77)	<.0001	<.0001
ALK							
Negative	299	1.0	0.88 (0.62, 1.25)	.4697	0.64 (0.44, 0.93)	.0199	.0211
Positive	29	1.0	1.12 (0.34, 3.68)	.8569	0.45 (0.06, 3.55)	.4477	.5637
Unknown	480	1.0	0.69 (0.53, 0.91)	.0088	0.55 (0.42, 0.71)	<.0001	<.0001
First‐line regiment							
Platinum‐based doublet chemotherapy	409	1.0	0.80 (0.59, 1.08)	.1404	0.62 (0.46, 0.82)	.0008	.0008
Single drug chemotherapy	36	1.0	1.27 (0.47, 3.41)	.6411	0.75 (0.24, 2.34)	.6187	.5822
Targeted therapy	125	1.0	0.77 (0.43, 1.38)	.3745	0.83 (0.50, 1.39)	.4804	.4800
Platinum‐based doublet chemotherapy plus angiogenesis‐therapy	49	1.0	1.34 (0.44, 4.05)	.6023	0.55 (0.20, 1.52)	.2487	.1697
Number of treatment lines							
≤3	488	1.0	0.74 (0.56, 0.98)	.0372	0.55 (0.42, 0.72)	<.0001	<.0001
>3	146	1.0	1.46 (0.85, 2.51)	.1733	1.17 (0.69, 1.97)	.5559	.5988

## 4 DISCUSSION

While some papers suggested a relationship between AAPR and patient prognosis in several types of cancer, this evidence in NSCLC is still scarce. Having additional markers for more accurate prognosis in patients with advanced NSCLC may help clinicians to decide on the best treatment regimen.

In the present study, we identified that decreased AAPR is associated with poor OS in patients with advanced NSCLC after adjusting other covariates, suggesting that AAPR may serve as a promising prognostic indicator in clinical practices.

Liver function test as an easily accessible and economically effective laboratory test has been widely employed in the routine clinical practice. Serum ALB and ALP levels are two important parameters of this test which can reflect biological and pathological changes under various illness conditions. Serum ALB is an important indicator that reflects the nutritional status of patients, as well as the inflammatory status of the body, and sometimes it even reflects the antitumor treatment response. A systematic review of 29 epidemiological studies showed that pretreatment ALB level is an independent predictor for patients’ survival, which is of great importance for evaluating the prognosis of cancer patients.^11^ Nine out of 10 studies focusing on the relationship between ALB and lung cancer also demonstrated that the higher ALB was positively correlated with the survival rate.^12^

Alkaline phosphatase dephosphorylates nucleotides, proteins, alkaloids, and other substrates. Although ALP is abundant in tissues and cells, its level in blood is usually very low. Under some pathological and specific physiological conditions such as pregnancy, bile duct obstruction, kidney disease, liver cancer, bone metastasis of malignant tumor, and other conditions, ALP level in serum will increase. Some studies identified that increased ALP level is correlated with some advanced cancer status.^13^, ^14^ Since Chan et al^15^ firstly reported the ratio of ALB to ALP combined with ALB and ALP levels can be used as an indicator for predicting the prognosis for patients with liver cancer, and such prediction ability is higher than that based on ALB or ALP levels alone, more studies began to investigate these indicators in other types of cancer.^7^, ^8^, ^9^, ^10^, ^16^, ^17^, ^18^, ^19^, ^20^, ^21^

Li et al^16^ firstly reported that the relationship between AAPR and OS after investigating 290 stage IV NSCLC patients, finding that AAPR was an independent predictor of OS in multivariate analysis (HR = 0.657, 95% CI = 0.504‐0.856, *P* < .01). Different from their study, we conducted a larger respective cohort as well as included more important variables for analysis, such EGFR and ALK mutation status, ECOG‐performance status, therapeutic regiment, and organ metastasis parameters, which have been verified to exert influence on the clinical outcome of the NSCLC patients. In a fully adjusted model, we identified that the groups with medium and high AAPR are associated with decreased 21% (HR = 0.79, 95%CI = 0.59‐1.06) and 41% (HR = 0.59, 95%CI = 0.44‐0.79) in risk of death as compared with low AAPR group with *P* trend .0003. The subgroup analysis also confirmed this stable and reproducible result. We also noted that inflection points of AAPR for predicting OS seems to be different for different tumor types. For example, Kim et al^10^ used inflection point 0.4876 for predicting clinical outcomes profiles (PFS, OS, etc) in patients with nonmetastatic nasopharyngeal carcinoma (NPC) before radical radiotherapy (RT). Ping Tan et al^17^ found that the lower AAPR was also an independent risk factor for poor OS in patients with upper tract urothelial carcinoma with inflection points 0.58. While as in lung cancer, Li et al^16^ identified that inflection point 0.36 for advanced NSCLC and Li et al^18^ used inflection points 0.61 for predicting OS in patients with limited small cell lung cancer. We speculate that the reasons for the different inflection points for AAPR may be in part due to the different patients’ groups. In consistence with the results of Li X and Ping Tan researches using 0.58 as cutoff value, we found that the no appropriative inflection point was detected in the smooth curve fitting. The relationship between AAPR and HR for OS seems not to be nonlinear, therefore we used tertile of AAPR for cutoff value alternatively.

Our study has several strengths: (a) to the best our knowledge, our sample size is relatively large compared with previous similar studies; (b) we firstly addressed the nonlinearity between AAPR and the risk of death in this study and explored this relationship further; (c) this study is an observational study and therefore susceptible to a potential confounding. We fully adjusted the potential covariates, which may influence the AAPR and OS to better elucidate the association between these parameters; (d) we used subgroup analysis as a sensitivity analysis to yield stable conclusion in different subgroups in this study.

However, several limitations in our study should be acknowledged. First, the single‐center property and a retrospective design are the major limitations. No independent cohorts were introduced to identify the prognostic value of AAPR. Second, the external validation of the prognostic value of AAPR is needed. Third, we just used pretreatment AAPR to predict the OS in patients with advanced NSCLC; whether the dynamic changes in the AAPR during the whole treatment course can predict the prognosis remains unknown.

In conclusion, our study indicates that AAPR can be an independent prognostic indicator in advanced NSCLC. The risk of death is negatively correlated with value of AAPR. A prospective study is required to validate the prognostic value of AAPR in those patients, and the mechanisms underlying the relationship between decreased AAPR and unfavorable survival in advanced NSCLC need to be further investigated.

## CONFLICT OF INTEREST

The authors declare no conflict of interest.

## AUTHOR CONTRIBUTIONS

Zhou Shaozhang involved in study design, funding support, statistical analysis, and paper writing. Jiang Wei and Wang Huilin involved in data collection, input, and statistical analysis. Wei Ni involved in data input and paper writing. Yu Qitao involved in study design, paper edition, and funding support.

## Data Availability

Some or all data used in the study are available from the corresponding author by request.
